# Effects of Sodium-Glucose Cotransporter-2 Inhibitors on Body Composition and Fluid Status in Cardiovascular Rehabilitation Patients with Coronary Artery Disease and Heart Failure

**DOI:** 10.3390/medicina60122096

**Published:** 2024-12-21

**Authors:** José C. De La Flor, Blanca Coto Morales, Elena Basabe, María Rey Hernandez, Rocío Zamora González-Mariño, Celia Rodríguez Tudero, Irwing Benites Flores, Carlos Espinoza, Michael Cieza Terrones, Secundino Cigarrán Guldris, Jesús Hernández Vaquero

**Affiliations:** 1 Department of Nephrology, Hospital Central de la Defensa Gómez Ulla, 28047 Madrid, Spain; jherva5@mde.es; 2 Department of Medicine and Medical Specialties, Faculty of Medicine, Alcala University, 28805 Madrid, Spain; 3 Health Sciences Doctoral Program, Faculty of Medicine, Alcala University, 28805 Madrid, Spain; 4 Department of Cardiology, Hospital Central de la Defensa Gómez Ulla, 28047 Madrid, Spain; bcotmor@mde.es (B.C.M.); mbasvel@mde.es (E.B.); mreyher@mde.es (M.R.H.); 5 Department of Nephrology, Hospital Universitario General Villalba, 28400 Madrid, Spain; rocio.zamora@quironsalud.es; 6 Department of Nephrology, Hospital Universitario de Salamanca, 37007 Salamanca, Spain; crodrigueztudero@usal.es; 7 PhD in Surgery Department, Faculty of Medicine, University of Salamanca, 37007 Salamanca, Spain; 8 Department of Nephrology, Hospital Alberto Barton Thompson, Lima 07001, Peru; irwing.benites@ibtgroup.com; 9 Faculty of Medicine, Peruana Cayetano Heredia University, Lima 15002, Peru; carlos.espinoza.m@upch.pe (C.E.); michael.cieza@upch.pe (M.C.T.); 10 Department of Nephrology, Hospital Ribera Polusa, 27004 Lugo, Spain; scigarran@riberacare.com

**Keywords:** sodium glucose cotransporter-2 inhibitors, bioimpedance analysis, type 2 diabetes mellitus, body composition, cardiovascular rehabilitation

## Abstract

*Background and Objectives*: Sodium glucose cotransporter-2 (SGLT-2) inhibitors have emerged as integral therapeutic tools in the management of patients with cardiovascular–kidney–metabolic (CKM) syndrome. In addition to their well-documented effects on lowering glucose levels and cardiovascular- and reno-protective actions, SGLT-2 inhibitors, through a reduction in body weight (BW), generate changes in the body composition and volume status that have not been clearly studied. *Materials and Methods*: This retrospective, observational longitudinal cohort, single-center study analyzed and compared body composition and fluid status measured by bioelectrical impedance analysis (BIA) from weeks 0 to 12 after the initiation of the cardiac rehabilitation (CR) program for coronary artery disease and heart failure in 59 patients who started treatment with SGLT-2 inhibitors (SGLT-2iG) and 112 patients without SGLT-2 inhibitors (non-SGLT-2iG). *Results:* Changes between the baseline and week 12 in the SGLT-2iG and non-SGLT-2iG were −0.3 L (*p* = 0.003) and −0.03 L (*p* = 0.82) in extracellular water (ECW) (*p* = 0.05), −0.39 L (*p* < 0.001) and −0.14 L (*p* = 0.33) in intracellular water (ICW) (*p* = 0.12), −0.69 (*p* < 0.001) and −0.16 (*p* = 0.52) in total body water (TBW) (*p* = 0.08), and −0.01 (*p* = 0.37) and −0.001 (*p* = 0.25) in the ECW/TBW ratio, respectively. After 3 months of exercise therapy in the CR program, patients in the SGLT-2iG showed a greater decrease than the non-SGLT-2iG in weight (−1.34 kg, *p* < 0.001 vs. −0.99, *p* = 0.02), body mass index (BMI) (−0.45 kg/m^2^, *p* < 0.001 vs. −0.38, *p* = 0.004), arm circumference (−0.57 cm, *p* = 0.008 vs. −0.12 cm, *p* = 0.21), waist circumference (−1.5 cm, *p* = 0.04 vs. −0.11 cm, *p* = 0.83), systolic blood pressure (SBP) (−8.9 mmHg, *p* = 0.049 vs. −4.19, *p* = 0.08), and diastolic blood pressure (DBP) (−5.15, *p* = 0.03 vs. −2.85, *p* = 0.01). The bioelectrical impedance analysis (BIA) revealed a significant decrease in body fat mass (BFM) and visceral fat area, without a loss of lean body mass (LBM) or skeletal muscle mass in the SGLT-2iG. *Conclusions*: SGLT-2 inhibitors exert beneficial effects on body compartments and volume status. Although they induce modest weight loss, this appears to be mainly directed at ECW, BFM, and visceral fat, without a loss of LBM nor skeletal muscle mass, which could contribute to the observed CKM benefits.

## 1. Introduction

Sodium glucose cotransporter-2 (SGLT-2) inhibitors have emerged as integral therapeutic tools in managing patients with overlapping cardiovascular disease (CVD), chronic kidney disease (CKD), and metabolic disorders such as type 2 diabetes mellitus (T2DM) and obesity [1,2], which was recently conceptualized as cardiovascular–kidney–metabolic (CKM) syndrome in stages 2, 3 and 4 [3,4]. Excess or dysfunctional adipose tissue is the main trigger of this CKM syndrome, causing insulin resistance, hyperglycemia, inflammation, oxidative stress, and vascular dysfunction, central processes that lead to the development of metabolic risk factors, progression of kidney disease, potentiation of heart–kidney interactions, and the development of CVD [4]. Thus, in addition to their well-documented effects on lowering glucose levels and cardiovascular- and reno-protective actions, SGLT-2 inhibitors, through a reduced body weight (BW), and changes in body composition [5] and volume status [6], may help control these events.

The effects of SGLT-2 inhibitors on weight loss are attributed to caloric loss (approximately 200–250 kcals daily) due to glucose excretion in urine [7]. Each of the available SGLT-2 inhibitors (empagliflozin, canagliflozin, and dapagliflozin) has been shown to reduce BW in diabetic patients, with canagliflozin achieving the greatest reduction in BW (between 1.8 and 3.4 kg) [8]. Additionally, SGLT-2 inhibitors have been shown to be effective at decreasing BW in individuals without diabetes. However, weight reduction in non-diabetics is usually lower than in diabetics, likely due to a smaller glycosuric effect [9]. Nevertheless, it is not clear which of the body compartments change following the BW reduction after the initiation of treatment with SGLT-2 inhibitors. Currently, there is very little evidence in this regard, but the weight loss caused by SGLT2 inhibitors is considered to generate changes in body compartments mainly associated with the loss of body fat mass (BFM) by a negative energy balance [5,10,11], decreasing lipid accumulation in visceral and subcutaneous fat, and reducing steatosis [12], or by the loss of sodium and extracellular volume [6,13], due to its recognized effect as an interstitial diuretic, or by a combination of these processes. However, some studies have shown that when BW is lost through energy restriction, not all the resulting weight loss can be attributed to BFM, as it is estimated that approximately 25% to 33% corresponds to a reduction in lean body mass (LBM) [14,15]. A large part of the LBM is made up of skeletal muscle; therefore, the losses of muscle mass and function can lead to sarcopenia, increasing the risks of falls, hospitalization, and physical frailty [16]. T2DM, sarcopenia, and obesity are intimately linked, and recently a new term “sarcopenic obesity” has been coined that is characterized by a low muscle mass, decreased muscle strength with physical dysfunction, and excess fat [17]. Consequently, although weight loss is an important goal in the treatment of CKM syndrome, it is important to understand the impact of new glucose-lowering treatments that promote weight loss, such as glucagon-like peptide-1 receptor agonists (GLP-1RAs) and SGLT-2 inhibitors, on body compartments.

On the other hand, cardiac rehabilitation (CR) is a personalized program designed to improve heart health that includes exercise training, emotional support, and heart-healthy lifestyle education. It is especially indicated in patients with coronary artery disease (CAD) or heart failure (HF) [18]. CR programs play a crucial role in cardiovascular care, being considered a Class IA recommendation to decrease the risks of future cardiovascular events and death from heart disease [19,20]. The combination of planned physical activity (aerobic and resistance exercise training in CR programs), dietary control, and integrated weight loss strategies prevent the development and progression of risk factors associated with CKM syndrome [21]. Aerobic exercise significantly reduces the risk of CVD, and strength/resistance exercise not only improves physical function but also lowers blood glucose levels and blood pressure [22].

During the process of the CR program, a noninvasive assessment of the body composition and fluid status are critical aspects for the routine evaluation, functional recovery, and long-term prognosis of patients with CKM syndrome [23]. The assessment of body composition at macroscopic levels can be divided into several compartmental models. The most used model in clinical practice is the 5-compartment model, which includes BFM, skeletal muscle mass, total body water (TBW), organs (these last three components are part of the LBM compartment), and bone (these last four components are part of the fat-free mass (FFM) compartment) [24]. It can be studied by direct (computed tomography (CT) and magnetic resonance imaging (MRI)) and indirect methods (bioelectrical impedance analysis (BIA), air displacement plethysmography (ADP) and dual-energy X-ray absorptiometry (DEXA)). Of the latter, BIA is the most used in clinical practice due to its low cost, speed, and easy reproducibility compared to CT, MRI, and DEXA.

BIA can contribute to identifying changes in body composition and fluid status during treatment with SGLT-2 inhibitors by influencing glucose and sodium homeostasis, which could have significant effects on these parameters, modulating BFM and LBM, as well as TBW. However, to our knowledge, it is not clear which of these body compartments changes after the introduction of SGLT-2 inhibitors. In this real-word data analysis, we aimed to investigate the effects of SGLT-2 inhibitors on body composition and fluid status in patients with CAD and HF participating in a CR program, specifically analyzing their impacts on the BFM, LBM, and TBW (intracellular water (ICW) and extracellular water (ECW)) distribution measured by BIA. This analysis seeks to clarify the potential roles of these agents in optimizing rehabilitation and improving the cardiovascular prognosis.

## 2. Materials and Methods

### 2.1. Study Design and Participants

This is a retrospective, observational longitudinal cohort, single-center study conducted in the Cardiac Rehabilitation Unit (CRU) of the Central Defense Hospital Gómez Ulla, in the period from January 2022 to January 2023. The inclusion criteria were as follows: (1) adult patients aged 18 years or older; (2) incident patients who were admitted to the CR program for HF and CAD, such as myocardial infarction, unstable angina, or coronary revascularization, procedures; (3) patients who had complete bioelectrical impedance analysis (BIA) data at admission and discharge from the CR program; and (4) patients who signed the informed consent form for participation in the study. Patients who did not complete the 3 months of the CR program; had been on SGLT-2 inhibitor treatment for more than 4 weeks prior to the start of the CR program; had incomplete BIA data; had a history of a clinical condition leading to changes in body composition, such as a malignant tumor, aggressive diet regimen, or bariatric surgery, or evidence of liver disease; had cognitive impairments and functional limitations; were pregnant; or declined to participate were excluded from the study.

**Figure 1:**
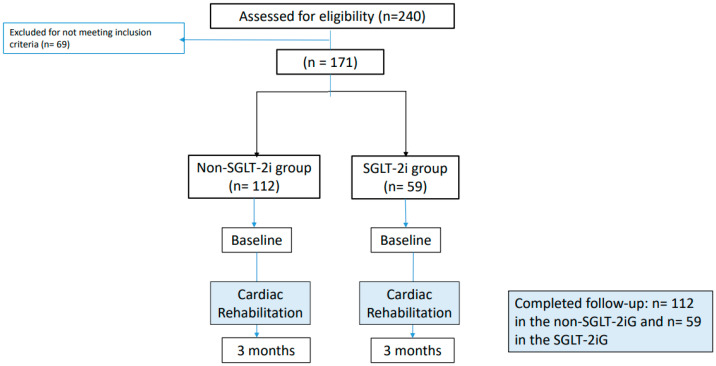
Flowchart of patient inclusion and exclusion criteria. Non-SGLT-2iG: group of patients treated without SGLT-2 inhibitors, SGLT-2iG: group of patients treated with SGLT-2 inhibitors.

The main indication for treatment with SGLT-2 inhibitors after the event of CAD was for their cardio-nephron-metabolic-protective effects, as indicated exclusively by the team of cardiologists. Of the 240 patients in the CR program, 171 met the inclusion criteria and were grouped into two groups: patients on SGLT-2 inhibitor treatment (SGLT-2iG) and patients not on SGLT-2 inhibitor treatment (non-SGLT-2iG) (Figure 1). Patients were included independently of the presence of diabetes and the type of SGLT-2 inhibitors (dapagliflozin, empagliflozin, or canagliflozin) prescribed.

The primary outcome of our study was to evaluate changes in the body composition and fluid status measured by BIA in patient with CAD and HF participating in the CR program who initiated treatment with SGLT-2 inhibitors compared with patients who did not receive SGLT-2 inhibitor treatment.

All participants provided written informed consent for the processing of the information obtained from their medical records, and all procedures were conducted in accordance with the Declaration of Helsinki after receiving ethical approval from the Clinical Research Ethics Committee of Hospital Central de la Defensa Gomez Ulla (Approval code number: 58/24).

### 2.2. Data Collection

Demographic data, the relevant clinical history, analytical data, and anthropometric variables of patients were obtained from their medical records at admission and discharge from the CR program. We calculated the body mass index (BMI) of all patients as their weight in kilograms divided by the square of their height in meters.

The analysis of the body composition and fluid status was performed using a direct segmental multifrequency BIA device with an eight-point tactile electrode system, with 30 impedance measurements taken using 6 frequencies (1, 5, 50, 250, 500, and 1000 kHz) at 5 segments (right arm, left arm, trunk, right leg, and left leg) (InBody S10. InBody Japan Inc., Tokyo, Japan). The measurement of the body fluid balance included TBW, ECW, ICW, and the ratio of ECW to TBW (ECW/TBW). These were calculated using the formula in the inner software based on the measured height, weight, and impedance [25]. The measurement of body composition included BFM, FFM, LBM, skeletal muscle mass, bone mineral mass, resistance, reactance, and phase angle (PA). The skeletal muscle index was calculated by dividing the appendicular skeletal muscle mass by the squared height (kg/m^2^). BIA measurements were taken before starting the first and last cardiac rehabilitation sessions in a supine decubitus position with no specific fasting or rest period. According to the InBody S10 user’s manual, the parameters of body composition and body fluid were calculated using a physiological calculation model: the body volume model for ICW, ECW, and TBW and the body composition model for LBM and BFM parameters [26].

### 2.3. Statistical Analysis

Continuous variables are shown as the means (standard deviations) or medians and interquartile ranges (IQRs), and categorical variables as valid percentages. We compared changes at 3 months as the difference between three-month and baseline data. Analytical parameters were compared between both SGLT-2iG and NSGLT-2G with Student’s *t* test (or the Mann–Whitney test for non-parametric data) or chi-square test, and with paired Student’s *t*-test when analyzing baseline data and at 3 months. The Kolmogorov–Smirnov test was used to determine whether the data had a normal distribution. To assess independent predictors of a decrease/increase in BFM or LBM, we used backward selected multivariable linear regression (pIN 0.05 pOUT 0.10). A *p*-value < 0.05 was considered statistically significant. The statistical package STATA 16.0 (Stata Statistical Software: Release 16. College Station, TX, USA: Stata Corp LP) was used for the statistical analyses.

## 3. Results

Out of the 240 patients who started the cardiac rehabilitation program during the study period, 171 patients were included in the study. A total of 71.4% were male, with a mean age of 66.9 (SD 11.2) years. Patients had a high percentage of comorbidities: 32.8% had T2DM, 65.5% had arterial hypertension (AHT), 14.6% had CKD, and 3.5% had chronic obstructive pulmonary disease (COPD). All of them had heart disease, mainly ischemic cardiomyopathy (ICM) (84.8%) and myocardiopathy (8.8%). In total, 13.5% had heart failure (HF). The mean left ventricular ejection fraction (LVEF) was 51.5% (SD 7.5%), with 10.5% classified as having a reduced EF (HFrEF), 15.2% as having a mildly reduced EF (HFmrEF), and 74.3% as having a preserved EF (HFpEF). A total of 9.9% had non-valvular atrial fibrillation (NVAF). All patients in the study underwent a stress test: ergometry (86%), ergo-spirometry (11.7%), or ergometry/ergospirometry (2.4%) (Table 1).

The population was classified into two groups, with 59 patients in the SGLT-2iG and 112 patients in the non-SGLT-2iG (Figure 1). In the group treated with SGLT-2 inhibitors, 39 (64.4%) patients were on treatment with empagliflozin, 19 (32.2%) patients with dapagliflozin, and 2 (3.4%) patients with canagliflozin. Baseline characteristics were not significantly different between both groups, except for the background of T2DM (SGLT-2iG 64% vs. non-SGLT-2iG 16.1%, *p* < 0.001), myocardiopathy (SGLT-2iG 23.7% vs. non-SGLT-2iG 0.9%, *p* < 0.001), HF (SGLT-2iG 30.5% vs. non-SGLT-2iG 4.5%, *p* < 0.001) and the estimated glomerular filtration rate (eGFR) by the chronic kidney disease epidemiology (CKD-EPI) creatinine equation (2021) (SGLT-2iG vs. non-SGLT-2iG 67.7 mL/min/1.73 m^2^). Patients in the SGLT2iG had a lower eGFR, although when divided by stage, the difference was not significant (*p* = 0.12). Most patients had an eGFR higher than 60 mL/min/1.73 m^2^. With respect to the procedures performed in the CR program, there were no differences in the type of exercise performed between the two groups. Adverse events from medication with the SGLT-2 inhibitors were reported by *n* = 3 patients (all urogenital infections).

After 3 months of training in the CR program, patients in the SGLT-2iG showed significantly reduced systolic and diastolic blood pressure (SBP/DBP), BW, BMI, waist circumference (WC), and arm circumference (AC) (Table 2).With respect to the control objectives of CR, both groups improved their exercise capacity, as measured by the metabolic equivalent of task (MET) (the ratio of the working metabolic rate to the resting metabolic rate) from 7.37 to 9.44, *p* < 0.001, in the non-SGLT-2iG and from 6.67 to 7.94, *p* < 0.001, in the SGLT-2iG. The blood sampling test results are presented in Table 2. During the trial period, no differences were observed between the groups, except for serum uric acid (SUA) levels and N-terminal prohormone of brain natriuretic peptide (NT-proBNP) levels. Furthermore, NT-proBNP levels improved significantly in the SGLT-2iG from 1810 (IQR: 220–3961) to 354.5 (IQR: 242–1482.5) (*p* < 0.001).

Table 3 and Table S1 present the changes in body composition and fluid status for both groups using the BIA InBodyS10 device at baseline and after 3 months of training in the CR program. The change in TBW was −0.16 L (*p* = 0.33) in the non-SGLT-2iG vs. −0.69 L (*p* < 0.001) in the SGLT-2iG, ICW was −0.14 L (*p* = 0.33) in the non-SGLT-2iG vs. −0.39 L in the SGLT-2iG (*p* < 0.001), and ECW was −0.03 L (*p* = 0.91) in the non-SGLT-2iG vs. −0.3 L (*p* = 0.85) in the SGLT-2iG. The change in the ECW/TBW ratio was 0.392 to 0.381 (*p* = 0.37) in the SGLT-2iG and 0.392 to 0.391 (*p* = 0.25) in the non-SGLT-2iG (Table 3). Compared with the patients in the non-SGLT-2iG, the SGLT-2iG showed a further significant reduction in ECW at 3 months (Table S1). On the other hand, the reduction in the percentage of patients with baseline fluid retention (according to an ECW/TBW ratio ≧ 0.40) was from 13 to 8% in the SGLT-2iG, with no significant differences (*p* = 0.1), while no differences were observed in the non-SGLT-2iG. Weight loss in the SGLT-2iG was not reflected in the loss of LBM (0.21 vs. −1.15 in non-SGLT-2iG, *p* = 0.03) nor skeletal muscle mass (−0.17 vs. −0.49 in non-SGLT-2iG, *p* = 0.13) in comparison with the non-SGLT-2iG (Table S1), but there was a statistically significant difference in terms of the reductions in waist circumference (from 95.42 cm to 93.92 cm, *p* = 0.04) and arm circumference (from 33.23 cm to 32.66 cm, *p* = 0.008), and although not reaching statistical significance, there was a decrease in the BFM (from 27.29 kg to 26.53 kg, *p* = 0.08) and visceral fat area (from 131.05 cm^2^ to 126.58 cm^2^, *p* = 0.08) (Table 3 and Figure S1).

The multivariate models generated did not identify SGLT-2 inhibitor treatment as an independent factor associated with BFM loss after adjusting for comorbidities and the type of exercise. We therefore conducted a sub-analysis to examine the differences between groups based on the presence or absence of T2DM. No significant differences were found when examining the analytical, anthropometric, and bioimpedance changes between patients with and without T2DM in each group (SGLT-2iG and non-SGLT-2iG) (Tables S2 and S3).

## 4. Discussion

To our knowledge, this study is the first with real-world data that evaluate the effects of SGLT-2 inhibitors on body composition and fluid status measured by a BIA device in patients with coronary artery disease and heart failure participating in a CR program. We observed that after 3 months of exercise therapy in the CR program, patients in the group treated with SGLT-2 inhibitors showed greater decreases in BW, BMI, AC, WC, and SBP/DBP compared to those who were not treated with SGLT-2 inhibitors. Second, SGLT-2iG patients showed significant reductions in SUA and NT-proBNP levels. Lastly, the reduction in BW during treatment with the SGLT-2 inhibitors was caused by changes in the fluid status, with decreases in ICW, ECW, and TBW, and by decreases in BFM and visceral fat area, without a loss of LBM or skeletal muscle mass during the following three months, which did not occur in patients without treatment with SGLT-2 inhibitors.

SGLT-2 inhibitors have emerged as a key treatment for patients with T2DM and CKD, and more recently, SGLT-2 inhibitors have become a main therapeutic pillar in the management of patients with HF, regardless of their LVEF. Natriuresis and osmotic diuresis mediated by SGLT-2 inhibition increases sodium delivery to the macula densa and leads to a rapid reduction in ECW, which inhibits renin production via the juxtaglomerular apparatus and triggers afferent vasoconstriction, decreasing the pressure in the glomerulus [27]. The impact of SGLT-2 inhibitors on the reduction in ECW is more pronounced than on ICW, which is congruent with their mechanism of action. Ohara K. et al. [28] conducted a study of forty CKD patients with fluid retention, and the patients were divided into three groups treated with an SGLT-2 inhibitor (dapagliflozin), loop diuretic (furosemide), or vasopressin V2 receptor antagonist (tolvaptan). The body fluid volume was measured on days 0 and 7 using a BIA device. BIA showed that the changes in ICW were similar but that there were significant changes in the ECW (dapagliflozin −8.4 ± 1.7, furosemide −12.5 ± 1.3, and tolvaptan −7.4 ± 1.5%, *p* = 0.048). This reduction in ECW is particularly beneficial in patients with HF, who often present volume overload and congestion. In many trials, such as EMPA-REG outcomes [13], EMPULSE [29] or EMPAG-HF [30], a significant improvement in clinical congestion was evident, supporting the hypothesis that a reduction in the ECW volume is a key component in the clinical benefits of SGLT-2 inhibitors. However, few studies have confirmed these findings through changes in the fluid status measured by BIA devices. One of the first studies discussing the effects of SGLT-2 inhibitors on the BIA-measured volume status was that of Schork A. et al. [5]. They conducted an observational longitudinal study and analyzed the body composition of 27 outpatients with T2DM during the first week and up to 6 months after the initiation of treatment with SGLT-2 inhibitors using a BIA device (Body Composition Monitor (BCM), Fresenius). At 6 months, BIA revealed that overhydration (OH) and ECW decreased by −0.5 L/1.73 m^2^ and −0.4 L/1.73 m^2^ at day 3, respectively, and returned to the initial values after 3 and 6 months, which were accompanied by the upregulation of the renin–angiotensin–aldosterone system (RAAS). Subsequently, Ohara K. et al. [31] performed a prospective study with 36 patients with diabetic kidney disease (DKD) who were treated with SGLT-2 inhibitors to evaluate the changes in the fluid status measured by a BIA device (InBodyS10) at baseline and day 7. They used the ECW/TBW ratio and ECW as markers of the extracellular volume status. The body weight, brain natriuretic peptide levels, and body fluid parameters measured by BIA (ICW, ECW, TBW, and ECW/TBW) were significantly decreased for 1 week after dapagliflozin administration. They concluded that the minor extracellular fluid-depleting effect of dapagliflozin on patients without severe extracellular fluid retention may contribute to maintaining an adequate body fluid status. Later, Schork A. et al. published two prospective observational studies to investigate whether SGLT-2 inhibitors might correct fluid overload in adult kidney transplant recipients (KTRs) (n = 22, follow up 6 months) [32] and in CKD patients (n = 42, follow up 6 months) [33]. The body composition and fluid status were measured by a BIA device (BCM, Fresenius). In both studies, Schork A. et al. concluded that SGLT-2 inhibitors reduce fluid overload in patients with elevated overhydration at baseline, while in euvolemic patients, the water status remained stable without a reduction in the body water content below the reference range, thus promoting the safety of the SGLT-2 inhibitor treatment. Recently, Mayne K., on behalf of the EMPA-KIDNEY Collaborative Group, conducted a sub-study with a BIA device (BCM, Fresenius) in approximately 10% of the main EMPA-KIDNEY trial population (660 patients) [34] that was recruited from centers in the UK and Germany to obtain measurements at baseline and after 2 and 18 months during follow-up. The sub-study was designed to assess changes in body composition, mainly fluid but also adiposity, to provide mechanistic information on the effects of empagliflozin. The primary outcome was the difference between the groups treated with empagliflozin (n = 332) or the placebo (n = 328) in the mean absolute fluid overload (AFO) during the follow-up period. The bioimpedance parameters of interest at randomization showed no statistically significant differences in AFO (empagliflozin 0.5 ± 1.7 L vs. placebo 0.3 ± 1.7) and relative fluid overload (RFO) (empagliflozin 1.9 ± 8.7% vs. placebo 1.3 ± 8.3%) between the groups. A weighted mean of the follow-up measurements adjusted to the baseline values was calculated and showed that, overall, the absolute value of fluid overload was 0.24 L lower in the empagliflozin group versus the placebo group, a highly statistically significant difference [6]. Their data were very similar to those found in our study: we observed a significantly greater reduction in ECW measured by a BIA device in the group of patients treated with SGLT-2 inhibitors, −0.3 (0.85) liters, compared to the group treated without SGLT-2 inhibitors, −0.03 (0.91) (*p* = 0.05). However, it is important to mention that the rate of mineralocorticoid receptor antagonist (MRA) use was significantly higher in the SGLT2-iG than in the non-SGLT2-iG (*p* < 0.001), thus justifying a higher diuretic action in the SGLT2-iG; unfortunately, the diuresis volume was not measured in the study groups. Initially, Mayne K. et al. [6] hypothesized that a greater difference would be observed in the 2-month period due to acute hemodynamic effects, but this does not appear to be the case consistently at both time points and was maintained for at least up to 18 months. The mean BW during the study period was 0.7 kg lower in patients who received empagliflozin vs. the placebo. This difference in weight was largely explained by differences in fluid, in particular ECW, which makes sense since the absolute fluid overload parameter largely consists of ECW. The authors found no statistically significant differences in changes in fat mass or lean mass between the two sub-study groups.

The effects of SGLT-2 inhibitors on ICW have been less studied, but some work, such as that of Hoshika Y. et al. [35], suggests that these drugs may have a relatively neutral effect on this parameter. They performed a subgroup analysis of the EMBODY trial [36], a prospective, randomized, double-blind, placebo-controlled trial of Japanese patients with acute myocardial infarction (AMI) and T2DM. Fifty-five patients underwent BIA using the device InBodyS10 (InBody Co., Ltd., Seoul, Republic of Korea) and were randomized to receive either 10 mg of empagliflozin or a placebo once daily for two weeks after the onset of AMI. They investigated changes in the volume status between weeks 0 and 24. In a stratified analysis, the rises in ECW and ICW were significantly attenuated in the empagliflozin group in contrast to the placebo group in participants with a BMI of 25 or higher but not in those with a BMI of less than 25. These results suggest that SGLT-2 inhibitors may affect the body fluid balance of obese patients. In our study, we also observed a non-significantly greater reduction in ICW between the baseline and the third month in the SGLT-2iG compared to non-SGLT-2iG, but no correlation with a high BMI. On the other hand, the ECW/TBW ratio is also considered a parameter of fluid balance, but its usefulness has been demonstrated for determining the dry weight of dialysis patients with HF assessed by the edema index [37]. In the study by Hoshika Y. et al. [35], no significant differences in the ECW/TBW ratio were observed between the empagliflozin and placebo groups. Similar data were observed in our study. Oka k. et al. [38] conducted a prospective, non-randomized, open-label study that included a dapagliflozin treatment group (n = 73) and a control group (n = 24) who were followed for 6 months. They observed that dapagliflozin decreased the ECW/TBW in patients with baseline fluid retention (ECW/TBW ≧ 0.400), but not in patients without baseline fluid retention (ECW/TBW < 0.400) (−1.47% ± 1.93% vs. −0.01% ± 1.88%, *p* = 0.0017). In our study, we observed only a non-significant reduction in the percentage of patients with baseline fluid retention from 13 to 8% in the SGLT-2iG (*p* = 0.1).

Several studies have reported that SGLT-2 inhibitors induce modest weight loss, which has been attributed primarily to the loss of BFM [5,10,11]. However, the precise composition of this loss has been the subject of debate. Body distribution studies using DEXA [10,11,39] and bioimpedance have demonstrated a reduction predominantly in BFM, especially in visceral adipose tissue (VAT) [8,40,41]. Schork et al. [5] found that the reduction in BW during treatment with SGLT-2 inhibitors is caused by changes in the volume status with a decrease in ECW during the first days of intake, and by a decrease in BFM during the following weeks and months. These data were very similar to those found in our study: we observed significantly greater reductions in BW (−1.34 kg vs. 0.99 kg) and BMI (0.45 vs. 0.38) in the SGLT-2iG compared to the non-SGLT-2iG. The BIA parameters showed non-significantly more pronounced reductions in the SGLT-2iG than in the non-SGLT-2iG with respect to BFM (−0.76 kg vs. −0.3 kg), body fat percentage (−0.61% vs. −0.13%), and visceral fat area (−0.57 cm^2^ vs. −0.12 cm^2^). However, in the EMPA-KIDNEY bioimpedance sub-study, they did not observe a significant effect of empagliflozin on the bioimpedance-derived adipose tissue mass (20.28 kg [95% CI 21.41–0.85]) [6]. In terms of BFM, studies that have directly compared SGLT-2 inhibitors with other weight loss interventions, such as GLP-1RA, suggest that although SGLT-2 inhibitors are less effective at reducing the total fat mass, visceral fat loss may be particularly significant.

On the other hand, the effects of medically induced weight loss on LBM and skeletal muscle mass have gained importance and significance in recent years due to data on the use of GLP-1RAs, which are effective for weight loss but can cause substantial muscle loss [42]. Skeletal muscle is the most abundant tissue in the human body, accounting for approximately 40% of the body mass and 25–30% of the basal energy expenditure [43]. Skeletal muscle is composed of 75% water, 20% protein, and 5% inorganic salts and minerals, and, on average, constitutes approximately 55% of FFM in BIA [44]. Given the importance of skeletal muscle for CKM health and physical function, Sargent JA. et al. [45] reviewed the available literature reporting the effects of GLP-1RAs and SGLT-2 inhibitors on body composition. The results demonstrate that, in most circumstances, the weight loss associated with both therapies predominantly involves a reduction in FBM, although significant heterogeneity exists between studies. In over half of the studies, they identified that the proportion of LBM reduction ranged between 20% and 50% of the total weight loss, which is consistent with diet-induced weight loss and bariatric surgery. The authors recommend paying more attention to the loss of LBM and skeletal muscle mass associated with GLP-1RA and SGLT-2-inhibitor-induced weight loss. In this regard, Pan R. et al. [46] conducted a systematic literature review and meta-analysis of 18 randomized controlled trials involving 1430 participants, of whom 726 received SGLT-2 inhibitors and 704 were controls. SGLT-2is were associated with significant improvements compared with other drugs in BW, BMI, WC, visceral fat area, subcutaneous fat area, FBM, and body fat percentage. However, SGLT-2 inhibitors were also associated with potentially adverse changes in body composition: LBM and skeletal muscle mass. The hypothesis put forward in this study is that SGLT-2 inhibitors induce skeletal muscle wasting by increasing the release of amino acids into the systemic circulation as a catabolic response to renal glucose excretion, preventing hypoglycemia. This study had several limitations, which generate low reliability of its results, such as the small number of trials that met the selection criteria, most trials having small sample sizes and heterogeneity in their study populations, short follow-up times (median, 24 weeks), and not analyzing changes in the ratio of LBM to BFM or changes in segmental lean mass. Two other trials involving 1737 participants with 52 to 104 weeks of follow-up showed similar data: although most of the SGLT-2-inhibitor-induced weight loss was due to a reduction in BFM, almost 10–30% was attributed to a reduction in LBM [47,48]. Recently, Wood et al. [49] conducted the first study to determine whether SGLT-2 inhibitors influence skeletal muscle pathology in patients with HFrEF. The analysis was based on previous studies in rodent models that showed that SGLT-2 inhibitors influenced skeletal muscle homeostasis [50,51]. Wood et al. [49] hypothesized that skeletal muscle pathology would be less in patients with HFrEF taking SGLT-2 inhibitors due to its anti-atrophic, pro-metabolic and anti-inflammatory effects. Muscle biopsies (pectoralis major) from 28 male patients with HFrEF treated with SGLT2is (>12 months) or without SGLT2is were compared. Comprehensive analyses of the muscle structure (immunohistochemistry), transcriptome (RNA sequencing), and metabolome (liquid chromatography–mass spectrometry) were performed, as well as serum inflammatory profiling (ELISA). The study found that treatment with SGLT-2 inhibitors in patients HFrEF was associated with lower rates of skeletal muscle pathology (myofiber atrophy was ~20% lower). The authors hypothesized that SGLT2is may attenuate muscle atrophy by reducing systemic IL-6 expression, which would lead to lower local IL-6 expression in skeletal muscle and ultimately reduce inflammation and atrophy. In agreement with our findings and one of the most relevant results of our study, patients in the SGLT-2iG did not present significant changes in skeletal muscle mass, skeletal muscle mass index (SMMI), or LBM. Very similar data were also observed in the EMPA-KIDNEY bioimpedance sub-study. In contrast, patients in the non-SGLT-2iG in our study did show significant reductions in these parameters (skeletal muscle mass, SMMI, LBM, and body cell mass (BCM)). In addition, the relative preservation of LBM with SGLT2is may be advantageous in certain patient populations, such as the elderly or those with chronic kidney disease, in whom sarcopenia and a loss of muscle mass may be important risk factors.

The phase angle (PA) is an additional parameter obtained from BIA. It is estimated by the direct ratio between resistance (R) and reactance (Xc). The PA can be considered as an indicator of fluid distribution between the ICW and ECW and may be an indicator of a malnutrition state. The PA, generally measured at 50 kHz by BIA, reflects the quality of the cells of the organism and has been related to the nutritional status of hemodialysis patients, with a phase angle of less than 4.5° indicating a malnourished patient [52]. However, in our study, when comparing the two groups, there was an increase of 0.03 (0.34) and a decrease of −0.07 (0.27) in the SGLT-2iG and non-SGLT-2iG, respectively, which was significant (*p* = 0.05).

SGLT-2 inhibitors have demonstrated beneficial effects on blood pressure (BP) in patients with and without T2DM and with other conditions such as HF and CKD [34,53,54]. SGLT-2 inhibitors reduce both SBP and DBP, probably through the osmotic diuresis and natriuresis they induce, which decrease the plasma volume and both preload and afterload [55]. Zhang et al. [56] systematically evaluated the clinical efficacy and safety of SGLT-2 inhibitors in patients with T2DM and hypertension by collecting previously published randomized controlled trials (RCTs) to provide data justifying the use of SGLT-2 inhibitors as an adjuvant in the first-line antihypertensive regimen in patients with T2DM and hypertension. RCTs comparing SGLT-2 inhibitors with the placebo in the treatment of T2DM with hypertension were selected. The primary efficacy endpoints were 24 h (H) SBP/DBP, and office SBP/DBP. Ten RCTs with 9913 participants (6293 in the SGLT-2 inhibitor group and 3620 in the control group) were included in the analysis. SGLT-2 inhibitors were effective at reducing BP compared to the placebo in patients with T2DM and hypertension. In our study, we observed a significant reduction in the in-office SBP and DBP after 3 months of SGLT-2 inhibitor treatment, in contrast to what we observed in the non-SGLT2-iG, where they only presented a significant reduction in the in-office DBP. The 24-h ambulatory SBP/DB was not measured.

In addition, these drugs have been observed to decrease SUA levels, which is attributed to increased renal excretion of this metabolite. This effect may have implications in reducing the risks of gout and other hyperuricemia-related disorders [57,58]. This latter effect of SGLT-2 inhibitors cannot be verified in the recent study published by Mayne K. on behalf of the EMPA-KIDNEY Collaborative Group [59]. They evaluated the effects of SGLT-2 inhibitors (empagliflozin) on uric acid (urate) levels and gout in patients with CKD. In the 6609 patients in the EMPA-KIDNEY study [34], the SUA levels were measured at randomization and then after 2 and 18 months of follow-up, and the effects of empagliflozin were analyzed using a pre-specified mixed model repeated measures approach. Participants that reported gout events were analyzed using Cox regression models (first events) with the Andersen–Gill extension (total events). The baseline mean SUA concentration ± SD was 431 ± 114 µmol/L. Compared to the placebo, empagliflozin did not significantly reduce first or total gout events (HR 0.87, 95%CI 0.74–1.02 for the 595 first events, and 0.86, 0.72–1.03 for the 869 total events). The authors concluded that SGLT-2 inhibition reduces SUA levels in patients with CKD, with greater effects at higher eGFR and in the absence of diabetes. However, the effect on SUA levels was modest and did not translate into a reduction in the gout risk in the EMPA-KIDNEY study [33]. Very similar data were observed in our study: patients in the SGLT-2iG presented a significantly greater reduction in SUA levels (5.1 (1.48) to 4.75 (1.26) mg/dL, *p* = 0.03) compared to patients in the non-SGLT-2iG (5.75 (1.63) to 5.65 (1.31) mg/dL, *p* = 0.3). During the follow-up period, patients in both groups did not present gout.

Natriuretic peptide, and specifically NT-proBNP levels, are used for the diagnostic and prognostic evaluation of HF, and several previous clinical trials have shown that there is possible heterogeneity in the response to treatment according to baseline NT-proBNP levels in patients with HFrEF or with HFpEF [60]. A post hoc analysis of the DELIVER study [61] evaluated the therapeutic effect of dapagliflozin as a function of baseline NT-proBNP levels in patients with HFrEF or HFpEF. Elevated NT-proBNP levels were part of the inclusion criteria (≥300 ng/L for non-atrial fibrillation or flutter (AFF); ≥600 ng/L for AFF) and the primary composite outcome was CV death and worsening HF. Among the 6262 patients included, the median baseline concentration of NT-proBNP was 716 (Q1-Q3: 469–1280) ng/L and 1399 (Q1-Q3: 962–2212) ng/L for patients with non-AFF and AFF, respectively. The clinical benefit of dapagliflozin was present irrespective of the baseline NT-proBNP concentration, and the absolute risk reduction was, therefore, greater in patients with higher NT-proBNP concentrations. In our study, we found similar data, a significant reduction in NT-proBNP levels in the SGLT-2iG of patients (1810 to 354.5 pg/Ml, *p* < 0.001). Recently, Chen et al. [60] performed a meta-analysis to assess the effects of SGLT-2 inhibitors on the disease-specific health status and cardiac function in chronic heart failure (CHF) patients. A total of 18 RCTs involving 23,953 participants were included in the meta-analysis. The effects of SGLT-2 inhibitors were compared with the control or placebo groups of patients with CHF with or without T2DM. The SGLT-2 inhibitor group exhibited a significant reduction in NT-proBNP levels of 136.03 pg/mL (95% confidence interval (CI): −253.36, −18.70; *p* = 0.02). Additionally, a greater proportion of patients in the SGLT-2 inhibitor group showed a ≥20% decrease in NT-proBNP levels (RR = 1.45, 95% CI (0.92, 2.29), *p* = 0.072). However, no statistically significant difference was observed for the effects on B-type natriuretic peptide (BNP). The use of SGLT-2 inhibitors led to a noteworthy improvement in LVEF by 2.79%. SGLT-2 inhibitors have shown beneficial effects on NT-proBNP levels in patients with HF, T2DM and CKD. These drugs have been observed to significantly reduce the levels of NT-proBNP, a marker reflecting volume overload and strain on the walls of the heart. This decrease may be due to the hemodynamic effects of SGLT2is, such as a reduced ventricular filling pressure and improved cardiac function, possibly related to natriuresis, osmotic diuresis, and the relief of congestion [60].

According to the Scientific Statement from the American Heart Association and the American Association of Cardiovascular and Pulmonary Rehabilitation of 2024 [62], weight management and body composition are essential components of the CR program. It is important to measure the relationship between BFM and LBM, as the latter is a more relevant indicator than BMI in the assessment of the cardiovascular risk. Simple assessments, such as WC, AC, and the waist-to-hip ratio, are effective and inexpensive methods for monitoring changes in body composition in routine clinical practice. In our study, we observed significantly greater reductions in AC (32.23 to 32.66 cm, *p* = 0.008 vs. 32.97 to 32.85 cm, *p* = 0.21) and WC (95.42 to 93.92 cm, *p* = 0.04 vs. 93.33 to 93.22, *p* = 0.83) in the SGLT-2iG of patients compared to the non-SGLT-2iG of patients, respectively. In addition, exercising for strength and building muscle is important for metabolic health. Skeletal muscle regulates more than 75% of insulin-mediated glucose disposal, which helps control glucose and lipids while reducing the risk of diabetes [63]. High-intensity aerobic training strategies and strength exercises are combined with dietary recommendations to improve both weight and body composition. MET is a measure used in exercise physiology to quantify the amount of energy expended during different physical activities. It represents the metabolic rate in relation to resting oxygen consumption. One MET equals the resting energy expenditure, that is, the number of calories the body burns while in a state of complete inactivity. One MET is equal to 3.5 milliliters of oxygen per kilogram of body weight per minute (mL/kg/min). In our study, we observed that the CR program had a significant impact on patients with coronary artery disease and heart failure in both groups in terms of subjective symptom perception on the MET scale. Patients in both groups presented a significant increase in the MET scale score, which was more pronounced in the non-SGLT-2iG and was probably related to a lower comorbidity compared to the SGLT-2iG of patients. To our knowledge, this is one of the first studies to evaluate the use of SGLT-2 inhibitors in patients undergoing the CR program.

Our study has several limitations that must be considered. The sample was small, and it is an observational study with a short follow-up time of 3 months, but the main limitation may be the disparity in the number of patients per group. On the other hand, our analysis is among the first to explore body composition and fluid status in the context of patients participating in a CR program who had coronary artery disease and heart failure. The use of BIA provides an objective method and results with parameters directly transferable into daily clinical application.

## 5. Conclusions

SGLT-2 inhibitors exert beneficial effects on body compartments and volume status that go beyond glycemic control. Although they induce modest weight loss, this appears to be mainly directed at FBM and visceral fat, without a loss of LBM or skeletal muscle mass during the following three months, which could contribute to the observed CKM benefits. In terms of the volume status, SGLT-2 inhibitors effectively reduce the extracellular volume, which is particularly beneficial in patients with heart failure, diabetic or non-diabetic kidney disease, and chronic kidney disease. These findings suggest that SGLT-2 inhibitors offer a unique clinical profile in the management of chronic diseases involving both metabolism and volume regulation.

## Figures and Tables

**Table 1:** Demographic characteristics according to the group.

	Non-SGLT-2iG	SGLT-2iG	Total	*p*-Value (Student’s *t*-Test)
n	112	59	171	
Age—years, mean (SD)	66 (11.34)	68.64 (10.87)	66.91 (11.2)	0.14
Sex—male, (%)	73.2	67.8	71.4	0.46
T2DM (%)	16.1	64.4	32.8	<0.001
AHTN (%)	65.2	66.1	65.5	0.9
CKD-EPI (mL/min/1.73 m^2^) (SD)	77.4 (18.4)	67.7 (21.4)	72.55	0.004
CKD-EPI <30 mL/min/1.73 m^2^ (%)	3.6	5.1	4.1	
CKD-EPI 30–60 mL/min/1.73 m^2^ (%)	13.4	25.4	17.5	0.12
CKD-EPI >60 mL/min/1.73 m^2^ (%)	83.0	69.5	78.4	
COPD (%)	3.6	3.4	3,5	0.95
Cardiomyopathy (%)	0.9	23.7	8,8	<0.001
Ischemic heart disease (%)	96.6	81.4	84.8	0.36
CHF (%)	4.5	30.5	13.5	<0.001
LVEF (%) (SD)	53.8 (3.4)	47.4 (10.7)	51.6 (7.5)	<0.001
LVEF (%)				<0.001
Preserved	83	57.6	74.3	
Mildly reduced	16.1	13.6	15.2	
Reduced	0.9	28.8	10.5	
NVAF (%)	7.1	15.3	9.9	0.09
Cardiac rehabilitation				
Stress test (%)				<0.001
Ergometry/Ergospirometry	0.89	5.1	2.4	
Ergometry	94.6	64.5	86	
Ergospirometry	4.5	25.4	11.7	
Rehabilitation risk (%)				0.001
Low–medium	41.3	14.6	32.3	
Medium	17.4	18.2	17.7	
High–medium	41.3	67.3	50	
Exercise (%)				0.08
Interval aerobic exercise	49.5	67.3	55.8	
Mixed aerobic exercise (intervallic and continuous)	3	3.6	3.2	
Continuous aerobic exercise	47.5	29.1	41	
Concomitant medication				
Sacubitril/Valsartan (%)	1.8	22	8.9	<0.001
RAASi (ACEis, ARBs) (%)	69.4	55.9	64.7	0.08
MRAs (%)				<0.001
Spironolactone	3.6	20.7	9.4	
Eplerenone	9.1	90.9	6.5	
Diuretics (%)				0.45
Hydrochlorothiazide	20.5	15.3	18.7	
Furosemide	9.8	15.3	11.7	
Torsemide	1.8	0	1.2	
GLP-1RA (%)	0.9	19	7.1	<0.001
SGLT-2is, n (%)				
Dapagliflozin	-	19 (32.2)		
Empaligflozin	-	38 (64.4)		
Canagliflozin	-	2 (3.4)		

Data are shown as means (standard deviations) or percentages (%). T2DM: type 2 diabetes mellitus, AHTN: arterial hypertension, CKD: chronic kidney disease; COPD: chronic obstructive pulmonary disease; CHF: congestive heart failure; LVEF: left ventricular ejection fraction; RAAS: renin–angiotensin–aldosterone system; ACEis: angiotensin-converting enzyme inhibitors; ARBs: angiotensin receptor blockers; MRAs: mineralocorticoid receptor antagonists; GLP-1RA: glucagon-like peptide-1 receptor agonist; SGLT-2is: sodium-glucose cotransporter-2 inhibitors.

**Table 2:** Changes in clinical data and in blood sample tests from baseline to three months.

	Non- SGLT2i Group		*p*-Value Paired *t*-Test	SGLT2i Group		*p* Value Paired *t*-Test
	Baseline (A)	Three Months (B)	A-B	Baseline (C)	Three Months (D)	C-D
Clinical measurements						
SBP (mmHg), mean (SD)	129.83 (19.57)	125.64 (12.88)	0.08	125.81 (22.07)	116.91 (24.21)	0.049
DBP (mmHg), mean (SD)	73.5 (10.86)	70.65 (8.19)	0.01	72.63 (12.34)	67.48 (9.57)	0.03
Weight (kg), mean (SD)	78.91 (16.49)	77.92 (16.06)	0.02	79.01 (14.13)	77.67 (13.8)	<0.001
BMI (kg/m^2^), mean (SD)	28.24 (5.28)	27.86 (5.07)	0.004	28.35 (4.31)	27.9 (4.17)	<0.001
Waist circumference (cm), mean (SD)	93.33 (12.58)	93.22 (12.9)	0.83	95.42 (15.26)	93.92 (14.01)	0.04
Arm circumference (cm), mean (SD)	32.97 (3.29)	32.85 (3.32)	0.21	33.23 (4.29)	32.66 (3.97)	0.008
METs, mean (SD)	7.37 (2.73)	9.44 (3.01)	<0.001	6.67 (2.58)	7.94 (2.79)	<0.001
Blood Sample Tests						
NT-proBNP (pg/mL), median [IQR]	235.5 [69.8–782]	218.5 [108–563]	0.69	1810 [220–3961]	354.5 [242–1482.5]	<0.001
Cr (mg/dL), mean (SD)	0.99 (0.32)	1 (0.29)	0.87	1.11 (0.36)	1.11 (0.41)	0.79
Na+ (mEq/L), mean (SD)	140.31 (2.4)	141.16 (1.96)	0.001	140.71 (1.97)	140.86 (1.76)	0.6
K+ (mEq/L), mean (SD)	4.47 (0.41)	4.51 (0.37)	0.45	4.61 (0.46)	4.68 (0.46)	0.2
Cl- (mEq/L), mean (SD)	103.93 (2.64)	104.5 (2.52)	0.06	103.32 (2.76)	103.8 (2.4)	0.21
Hb (g/L), mean (SD)	14.21 (1.71)	15.55 (14.67)	0.72	14.39 (1.62)	14.36 (1.52)	0.65
Serum uric acid (mg/dL), mean (SD)	5.75 (1.63)	5.65 (1.31)	0.3	5.1 (1.48)	4.75 (1.26)	0.03
ACRU (mg/g), mean (SD)	68.31 (29.02)	49.89 (37.55)	0.81	56.32 (42.44)	42.83 (34.71)	0.35

Data are shown as means (SDs), medians [interquartile ranges—IQRs], or numbers (percentages). SBP: systolic blood pressure; DBP: diastolic blood pressure; BMI: body mass index; METs: metabolic equivalents; NT-proBNP: N-terminal prohormone of brain natriuretic peptide; Cr: creatinine; Na+: serum sodium; K+: serum potassium; Cl-: chloride, Hb: hemoglobin; HbA1c: glycosylated hemoglobin; ACRU: albumin-to-creatinine ratio in urine.

**Table 3:** Bioimpedance data at baseline and 3 months after rehabilitation, according to the groups.

	Non-SGTL2i Group		*p*-Value Paired t-Test	SGLT2i Group		*p*-Value Paired t-Test
	Baseline (A)	Three Months (B)	A-B	Baseline (C)	Three Months (D)	C-D
Bioimpedance						
TBW (L), mean (SD)	37.99 (6.99)	37.83 (6.87)	0.52	38.84 (7.17)	37.9 (7.52)	<0.001
ICW (L), mean (SD)	23.28 (4.36)	23.15 (4.27)	0.33	23.88 (4.57)	23.49 (4.48)	<0.001
ECW (L), mean (SD)	14.71 (2.68)	14.68 (2.65)	0.82	14.96 (2.66)	14.66 (2.6)	0.003
ECW/TBW ratio, mean (SD)	0.392 (0.013)	0.391 (0.013)	0.25	0.392 (0.011)	0.381 (0.011)	0.37
Protein (Kg), mean (SD)	10.32 (1.97)	10.16 (1.93)	<0.001	10.07 (1.88)	10 (1.83)	0.25
Minerals (Kg) mean (SD)	3.65 (0.68)	3.56 (0.64)	<0.001	3.56 (0.64)	3.56 (0.63)	0.84
BFM (kg), mean (SD)	26.2 (10.08)	25.9 (10.03)	0.26	27.29 (11.31)	26.53 (10.75)	0.08
FFM (kg), mean (SD)	52.8 (9.82)	51.86 (9.58)	<0.001	51.61 (9.5)	51.38 (9.31)	0.5
Percent body fat (%), mean (SD)	32.64 (9.4)	32.77 (9.47)	0.66	33.66 (9.36)	33.06 (8.79)	0.21
Visceral fat area (cm^2^), mean (SD)	121.62 (50.83)	121.04 (51.84)	0.71	131.05 (57.2)	126.58 (52.73)	0.08
Bone mineral content (kg), mean (SD)	3 (0.56)	3.2 (2.87)	0.46	2.94 (0.54)	2.94 (0.52)	0.92
Skeletal muscle mass (kg), mean (SD)	29.14 (5.96)	28.64 (5.84)	<0.001	28.35 (5.71)	28.18 (5.56)	0.37
LBM (kg), mean (SD)	49.81 (9.25)	48.66 (9.47)	0.002	48.23 (8.95)	48.44 (8.8)	0.72
BTM (kCals/24 h), mean (SD)	1510.31 (211.86)	1490.25 (206.9)	<0.001	1484.85 (205.25)	1479.86 (201.05)	0.5
BCM (kg), mean (SD)	34.21 (6.55)	33.56 (6.51)	<0.001	33.31 (6.29)	33.14 (6.11)	0.41
TBW/FFM (%), mean (SD)	73.57 (0.4)	73.57 (0.36)	0.95	73.61 (0.37)	72.87 (5.62)	0.32
SMMI (kg/m^2^), mean (SD)	7.97 (1.03)	8.42 (6.33)	<0.001	7.75 (1.11)	7.69 (1.09)	0.28
Phase angle	5.59 (0.88)	5.62 (0.87)	0.3	5.41 (0.72)	5.35 (0.69)	0.06

Data are shown as means (SDs), medians [interquartile ranges—IQRs], or numbers (percentages). TBW: total body water; ICW: intracellular water; ECB: extracellular water; BFM: body fat mass; FFM: fat-free mass; LBM: lean body mass; BMT: basal metabolic rate; h: hours; BCM: body cell mass; SMMI: skeletal muscle mass index.

## Data Availability

No new data were created or analyzed in this study. The data used to support the findings of this study are available from the corresponding author upon request (contact J.C.D.L.F., jflomer@mde.es). We confirm that all the figures and tables are the original work of this manuscript’s authors. The authors of this manuscript have created it all, which has not been adapted from other authors, and do not present an online link.

## Supplementary Materials

The following supporting information can be downloaded at https://www.mdpi.com/article/10.3390/medicina60122096/s1: Table S1. BIA-measured changes in body composition and fluid status at baseline and after three months of training, showcasing the profound impact of the CR program on these metrics; Table S2. Changes in the clinical data, blood test results, and bioimpedance from baseline to three months in patients who were not treated with SGLT-2 inhibitors, divided by the presence or absence of diabetes mellitus; Table S3. Changes in the clinical data, blood test results, and bioimpedance from baseline to three months in patients who were treated with SGLT-2 inhibitors, divided by the presence or absence of diabetes mellitus; Figure S1. BIA-measured changes in body composition according to the groups (SGLT-2i-treated and non-SGLT 2i-treated patients) during follow-up in the cardiac rehabilitation (CR) program.
