# Supplementary material for: Effects of Sodium-Glucose Cotransporter-2 Inhibitors on Body Composition and Fluid Status in Cardiovascular Rehabilitation Patients with Coronary Artery Disease and Heart Failure
Source: Medicina (Kaunas). 2024 Dec 21;60(12):2096. doi: 10.3390/medicina60122096 (PMC11677857; doi:10.3390/medicina60122096)
Supplement: Supplementary file 1 [file medicina-60-02096-s001.zip › medicina-3358703-supplementary.pdf]

Table S1. BIA-measured changes in body composition and fluid status at baseline and after three months of training, showcasing the profound impact of the CR program on these metrics.

.	Non-SGLT-2i group	SGLT-2i group	p-value
	**dif A-B (E)**	**dif B-C (F)**	**e-f**
**Bioimpedance measured**			
TBW (L), dif. mean	-0.16 (1.93)	-0.69 (1.7)	0.08
ICW (L), dif. mean	-0.14 (1.07)	-0.39 (0.95)	0.12
ECW (L), dif. mean	-0.03 (0.91)	-0.30 (0.85)	0.05
Protein (kg), dif. mean	-0.17 (0.43)	-0.07 (0.46)	0.19
Minerals (kg), dif. mean	-0.08 (0.23)	0.01 (0.26)	0.03
BFM (kg), dif. mean	-0.30 (2.82)	-0.76 (3.3)	0.34
FFM (kg), dif. mean	-0.94 (2.61)	-0.23 (2.6)	0.09
Percent body fat (%), dif. mean	0.13 (3.24)	-0.61 (3.7)	0.18
Visceral fat area (cm^2), dif. mean	-0.12 (1)	-0.57 (1.59)	0.17
Bone mineral content (kg), dif. mean	0.20 (2.82)	0 (0.23)	0.59
Skeletal muscle mass (kg), dif. mean	-0.49 (1.25)	-0.17 (1.44)	0.13
LBM (kg), dif. mean	-1.15 (3.82)	0.21 (4.16)	0.03
BTM (kCals/24 h), dif. mean	-20.06 (49.51)	-4.98 (56.4)	0.07
BCM (kg), dif. mean	-0.57 (16.6)	-4.47 (19.56)	0.068
Arm circumference (cm), dif. mean	-0.65 (1.62)	-0.17 (1.58)	0.03
Waist circumference (cm), dif. mean	-0.11 (5.07)	-1.50 (5.56)	0.10
TBW/FFM (%), dif. mean	0 (0.28)	-0.73 (5.63)	0.17
SMMI (kg/m2), dif. mean	-0.14 (0.35)	-0.05 (0.36)	0.11
Phase angle	0.03 (0.34)	-0.07 (0.27)	0.05

Data are shown as mean (SD) or number (percentage). TBW: total body water; ICW: intracellular water; ECB: extracellular water; BFM: Body fat mass; FFM: Fat-free mass; LBM: lean body mass; BMT: basal metabolic rate; h: hours; BCM: Body cell mass; SMMI: skeletal muscle mass index.

Table S2. Changes in the clinical data, blood test results, and bioimpedance from baseline to three months in patients who were not treated with SGLT-2 inhibitors, divided by the presence or absence of diabetes mellitus.

	Non-SGLT2i Group					
	Non-T2DM			T2DM		
	Baseline (A)	Three months (B)	A-B	Baseline (C)	Three months (D)	C-D
	PRE	POST		PRE	POST	
**N**	94			18		
**Clinical measurements**						
SBP (mmHg), mean (SD)	128.13 (17.98)	124.89 (12.34)	0.23	137.53 (24.75)	129.27 (15.2)	0.19
DBP (mmHg), mean (SD)	74.17 (10.72)	71.14 (7.82)	0.01	70.47 (11.33)	68.27 (9.78)	0.64
Weight (kg), mean (SD)	75.55 (13.21)	73.8 (12.31)	0.09	80.76 (17.94)	80.19 (17.53)	0.05
BMI (kg/m2), mean (SD)	27.9 (4.37)	27.22 (4.14)	0.04	28.43 (5.77)	28.22 (5.54)	0.04
Waist circumference (cm), mean (SD)	93.87 (12.08)	93.56 (12.45)	0.55	90.49 (15.02)	91.44 (15.3)	0.47
Arm Circumference (cm), mean (SD)	33.2 (3.2)	33.02 (3.25)	0.09	31.79 (3.61)	31.96 (3.63)	0.51
METs, mean (SD)	7.47 (2.79)	9.57 (3.1)	<0.001	6.77 (2.35)	8.65 (2.34)	0.005
**Blood Sampling Test**						
NT-proBNP (pg/mL), median [IQR]	168 [58.3-350]	153 [78.2-437.5]	0.78	981 [677-1069]	1598.5 [283-2914]	-
Cr (mg/dl), mean (SD)	1 (0.32)	1 (0.28)	0.9	0.96 (0.37)	0.97 (0.38)	0.84
Na+ (mEq/L), mean (SD)	140.23 (2.4)	141.08 (1.95)	0.004	140.76 (2.44)	141.65 (2)	0.16
K+ (mEq/L), mean (SD)	4.47 (0.41)	4.53 (0.38)	0.32	4.5 (0.45)	4.44 (0.36)	0,49
Cl- (mEq/L), mean (SD)	103.93 (2.54)	104.57 (2.3)	0.06	103.88 (3.22)	104.06 (3.57)	0.82
Hb (g/L), mean (SD)	14.35 (1.68)	14.33 (1.45)	0.7	13.48 (1.71)	13.47 (1.37)	0.99
Serum uric acid (mg/dl), mean (SD)	5.73 (1.61)	5.6 (1.29)	0.24	5.9 (1.75)	5.95 (1.42)	0.84
ACRU (mg/g), mean (SD)	68.58 (27.92)	48.41 (37.44)	0.95	67.34 (34.02)	57.56 (38.82)	0.42
**Bioimpedance**						
TBW (L), mean (SD)	35.26 (5.85)	35.34 (5.27)	0.86	39.5 (7.18)	39.2 (7.31)	0.32

ICW (L), mean (SD)	21.6 (3,6)	21.59 (3.24)	0.62	24.21 (4.5)	24.01 (4.56)	0.5
ECW (L), mean (SD)	13.66 (2.31)	13.76 (2.12)	0.94	15.29 (2.73)	15.19 (2.79)	0.22
ECW/TBW ratio, mean (SD)	0.39 (0.01)	0.39 (0.01)	0.14	0.39 (0.01)	0.39 (0.01)	0.69
Protein (Kg), mean (SD)	10.39 (2.05)	10.23 (2.02)	<0.001	9.98 (1.48)	9.78 (1.38)	0.047
Minerales (Kg) mean (SD)	3.65 (0.7)	3.58 (0.67)	0.06	3.61 (0.56)	3.47 (0.5)	0.02
BFM (kg), mean (SD)	26.32 (10.02)	25.88 (9.98)	0.15	25.57 (10.66)	25.96 (10.55)	0.52
FFM (kg), mean (SD)	53.01 (10.22)	52.17 (9.99)	0.003	51.72 (7.53)	50.29 (7.05)	0.01
Percent body Fat (%), mean (SD)	32.8 (9.5)	32.8 (9.6)	0.97	31.96 (9.0)	32.9 (9.3)	0.23
Visceral fat area (cm^2), mean (SD)	122.11 (51.1)	120.86 (51.88)	0.47	119.02 (50.79)	122.01 (53.12)	0.4
Bone mineral content (kg), mean (SD)	3.01 (0.58)	3.27 (3.13)	0.4	2.98 (0.47)	2.88 (0.42)	0.03
Skeletal muscle mass (kg), mean (SD)	29.33 (6.21)	28.87 (6.09)	<0.001	28.12 (4.51)	27.45 (4.19)	0.02
LBM (kg), mean (SD)	50.01 (9.63)	49.22 (9.46)	<0.001	48.74 (7.09)	45.76 (9.23)	0.14
BTM (kCals/24 h), mean (SD)	1514.75 (220.46)	1496.75 (215.87)	<0.001	1487.17 (162.79)	1456.33 (152.04)	0.02
BCM (kg), mean (SD)	34.42 (6.81)	33.79 (6.81)	<0.001	33.08 (4.97)	32.33 (4.6)	0.02
TBW/FFM (%), mean (SD)	73.54 (0.4)	73.55 (0.34)	0.81	73.72 (0.39)	73.67 (0.45)	0.28
SMMI (kg/m2), mean (SD)	7.96 (1.06)	7.84 (1.08)	<0.001	8.01 (0.9)	7.78 (0.74)	0.024
Phase angle	5.69 (0.85)	5.72 (0.84)	0.45	5.04 (0.85)	5.1 (0.9)	0.31

Data are shown as mean (SD) or median [interquartile range—IQR] or number (percentage). SBP: Systolic blood pressure; DBP: Diastolic blood pressure; BMI: Body mass index; METs: metabolic equivalents; NT-proBNP: N-terminal prohormone of brain natriuretic peptide; Cr: Creatinine; Na+: serum sodium; K+: serum potassium; Cl-: chloride, Hb: Hemoglobin; HbA1c: Glycosylated hemoglobin; ACRU: Albumin to Creatinine Ratios Urine. TBW: total body water; ICW: intracellular water; ECB: extracellular water; BFM: Body fat mass; FFM: Fat free mass; LBM: lean body mass; BMT: basal metabolic rate; h: hours; BCM: Body cell mass; SMMI: skeletal muscle mass index.

Table S3. Changes in the clinical data, blood test results, and bioimpedance from baseline to three months in patients who were treated with SGLT-2 inhibitors, divided by the presence or absence of diabetes mellitus.

	SGLT2i Group					
	No DM			DM		
	**Baseline (E)**	**Three months (F)**	**E-F**	**Baseline (G)**	**Three months (H)**	**G-H**
	**PRE**	**POST**		**PRE**	**POST**	
**N**	**21**			**38**		
**Clinical measurements**						
SBP (mmHg), mean (SD)	115.53 (16.33)	106.81 (31.32)	0.26	130.95 (22.93)	122.89 (16.76)	0.1
DBP (mmHg), mean (SD)	68.89 (10.86)	63.07 (8.1)	0.19	74.5 (12.74)	69.93 (9.57)	0.08
Weight (kg), mean (SD)	79.34 (13.86)	77.94 (13.59)	<0.001	77.29 (15.76)	76.25 (15.23)	0.04
BMI (kg/m^2), mean (SD)	28.39 (4.22)	27.94 (4.12)	<0.001	28.1 (4.88)	27.69 (4.58)	0.04
Waist circumference (cm), mean (SD)	94.66 (12.42)	91.57 (10.33)	0.03	95.84 (16.77)	95.22 (15.65)	0.47
Arm Circumference (cm), mean (SD)	32.61 (3.45)	31.7 (2.92)	0.01	33.57 (4.69)	33.19 (4.39)	0.17
METs, mean (SD)	6.23 (2.69)	6.94 (3.43)	0.29	6.81 (2.57)	8.26 (2.54)	0.001
**Blood Sampling Test**						
NT-proBNP (pg/mL), median [IQR]	920 [371-1572]	1810 [968-2399.5]	0.28	308 [147-925]	2090.5 [73.5-5051]	0.49
Cr (mg/dl), mean (SD)	1.13 (0.39)	1.15 (0.42)	0.63	1.09 (0.35)	1.09 (0.41)	0.99
Na^+ (mEq/L), mean (SD)	140.76 (1.61)	140.9 (1.58)	0.68	140.68 (2.16)	140.84 (1.87)	0.7
K^+ (mEq/L), mean (SD)	4.6 (0.34)	4.62 (0.38)	0.73	4.62 (0.52)	4.71 (0.51)	0.2
Cl^- (mEq/L), mean (SD)	104.19 (2.18)	103.95 (1.91)	0.61	102.84 (2.95)	103.71 (2.65)	0.1
Hb (g/L), mean (SD)	14.34 (1.79)	14.6 (1.26)	0.29	14.41 (1.53)	14.22 (1.65)	0.12
Serum uric acid (mg/dl), mean (SD)	5.35 (1.62)	4.51 (1.16)	0.04	4.95 (1.4)	4.88 (1.31)	0.31
ACRU (mg/g), mean (SD)	61.13 (52.35)	54.59 (39.42)	0.34	53.03 (35.11)	37.63 (31.67)	0.69
**Bioimpedance**						
TBW (L), mean (SD)	38.98 (7.46)	38.38 (7.37)	<0.001	38.13 (5.53)	37.01 (5.23)	0.01
ICW (L), mean (SD)	24.03 (4.75)	23.67 (4,68)	0.004	23.11 (3.47)	22.58 (3.22)	0.02

ECW (L), mean (SD)	14.94 (2.76)	14.69 (2.69)	<0.001	15.03 (2.12)	14.48 (2.08)	0.02
ECW/TBW ratio, mean (SD)	0.38 (0.01)	0.38 (0.01)	0.71	0.39 (0.01)	0.39 (0.01)	0.18
Protein (Kg), mean (SD)	9.34 (1.56)	9.32 (1.39)	0.87	10.47 (1.94)	10.37 (1.96)	0.16
Minerales (Kg) mean (SD)	3.32 (0.53)	3.36 (0.45)	0.56	3.69 (0.67)	3.68 (0.68)	0.853
BFM (kg), mean (SD)	27.63 (9.11)	25.78 (8.48)	0.04	27.1 (12.47)	26.94 (11.9)	0.73
FFM (kg), mean (SD)	47.92 (7.91)	48 (7.1)	0.89	53.65 (9.78)	53.25 (9.93)	0.33
Percent body Fat (%), mean (SD)	35.8(8.7)	34.1 (8.5)	0.06	32.5(9.6)	32.5(9.0)	0.97
Visceral fat area (cm^2), mean (SD)	137.05 (51.04)	125.61 (46.44)	0.045	127.74 (60.73)	127.12 (56.49)	0.8
Bone mineral content (kg), mean (SD)	2.74 (0.44)	2.77 (0.37)	0.63	3.05 (0.56)	3.04 (0.57)	0.8
Skeletal muscle mass (kg), mean (SD)	26.19 (4.7)	26.15 (4.23)	0.91	29.55 (5.92)	29.31 (5.93)	0.28
LBM (kg), mean (SD)	45.18 (7.49)	45.25 (6.72)	0.9	49.92 (9.33)	50.2 (9.38)	0.72
BTM (kCals/24 h), mean (SD)	1404.91 (171.07)	1406.95 (152.95)	0.88	1529.03 (211.15)	1520.16 (214.55)	0.32
BCM (kg), mean (SD)	30.96 (5.17)	30.91 (4.64)	0.9	34.61 (6.53)	34.37 (6.52)	0.33
TBW/FFM (%), mean (SD)	73.57 (0.41)	73.6 (0.42)	0.54	73.62 (0.34)	72.47 (7)	0.32
SMMI (kg/m^2), mean (SD)	7.35 (1.01)	7.35 (0.89)	0.95	7.97 (1.12)	7.88 (1.16)	0.18
Phase angle	5.24 (0.72)	5.13 (0.71)	0.09	5.51 (0.71)	5.47 (0.67)	0.32

Data are shown as mean (SD) or median [interquartile range—IQR] or number (percentage). SBP: Systolic blood pressure; DBP: Diastolic blood pressure; BMI: Body mass index; METs: metabolic equivalents; NT-proBNP: N-terminal prohormone of brain natriuretic peptide; Cr: Creatinine; Na+: serum sodium; K+: serum potassium; Cl-: chloride, Hb: Hemoglobin; HbA1c: Glycosylated hemoglobin; ACRU: Albumin to Creatinine Ratios Urine. TBW: total body water; ICW: intracellular water; ECB: extracellular water; BFM: Body fat mass; FFM: Fat free mass; LBM: lean body mass; BMT: basal metabolic rate; h: hours; BCM: Body cell mass; SMMI: skeletal muscle mass index.

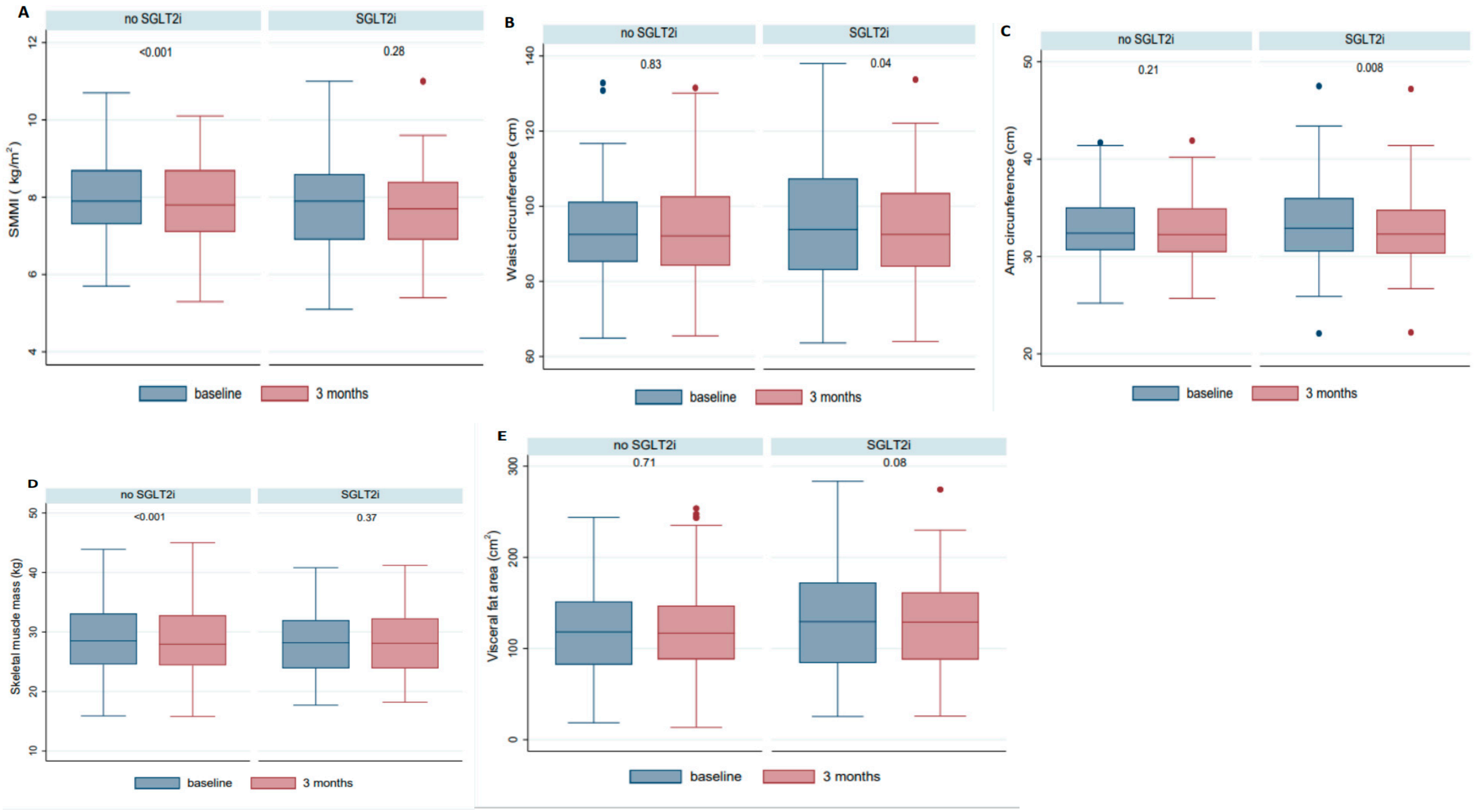

Figure S1. BIA-measured changes in body composition according to the groups (SGLT-2i treated and non-SGLT 2i treated patients) during follow-up in the cardiac rehabilitation (CR) program. Changes in SMMI (skeletal muscle mass index) (kg/m2) **(A)** waist circumference (cm); **(B)** arm circumference (cm); **(C)** skeletal muscle mass (kg); **(D)** and visceral fat area (cm^2); **(E)** compared at baseline with third month of CR program in patients with and without treatment with SGLT-2 inhibitors, Box plots show medians with thick lines.
